# A Case Report of Kissing Carotid Arteries in the Retropharynx

**DOI:** 10.22038/IJORL.2022.58891.3033

**Published:** 2022-05

**Authors:** Siti Asmat Md Arepen, Azreen Zaira Abu Bakar, Nour Hanan Daniah Mohd Bakhit, Ahmad Anwaar bin Muhammad Saifullah, Nor Azirah Salahuddin, Nor Eyzawiah Hassan

**Affiliations:** 1 *Otorhinolaryngology Head and Neck Surgery, Surgical-based Department, Faculty of Medicine and Health Sciences, University Sains Islam Malaysia, Negeri Sembilan, Malaysia.*; 2 *Department of Otorhinolaryngology, Hospital Kuala Lumpur, Ministry of Health, Malaysia.*

**Keywords:** Arterial tortuosity, Life-threatening, Retropharyngeal, Transoral surgeries.

## Abstract

**Introduction:**

An aberrant carotid artery has distinct terms and may exhibit a submucosal mass in the posterior pharyngeal wall. While it is primarily asymptomatic, an extreme aberrancy doubles the risk of dissection, a cerebrovascular accident (CVA) and an injury intraoperatively.

**Case Report:**

We report a case of ‘kissing carotid artery’ in a 65-year-old lady who presented with a foreign body sensation felt in the throat for one week. A finding of flexible nasopharyngolaryngoscopy (FNPLS) showed a bilateral paramedian retropharyngeal pulsating mass. Radiological examinations, including Computed Tomography (CT) of the neck and an angiogram, revealed an aberrant course of bilateral carotid arteries. In view of no malignancy and vascular malformations, there was no further intervention done, and the patient was subjected to yearly surveillance.

**Conclusion:**

Retropharyngeal carotid arteries are clinically significant anatomic variants. Such anomalies are potentially life-threatening and a risk factor for a severe hemorrhage during the simplest and commonly performed transoral surgeries. Thus, thorough perioperative assessment with accurate imaging techniques and studies are required to evaluate these anomalies and may avoid any disastrous complications.

## Introduction

Retropharyngeal internal carotid arteries (ICA) are relatively rare, with an incidence of 2.6% to 10%, but they are well-described anatomic variants relevant to transoral approach surgeries (1). A variation of its common pathway may be placed in a retropharyngeal position adjacent to the posterior pharyngeal wall. We herein report a case of an aberrant course of the bilateral carotid artery presented as a ‘kissing carotid artery’ and its clinical importance and risks.

## Case Report

A 62-year-old lady with no known medical illness was presented with a foreign body sensation felt in the throat for one week associated with a bad-smelling breath. She denied any odynophagia, dysphagia, fever, and any foreign body ingestion, change of voice or stridor. There was no history of trauma or fall. 

**Fig 1:**
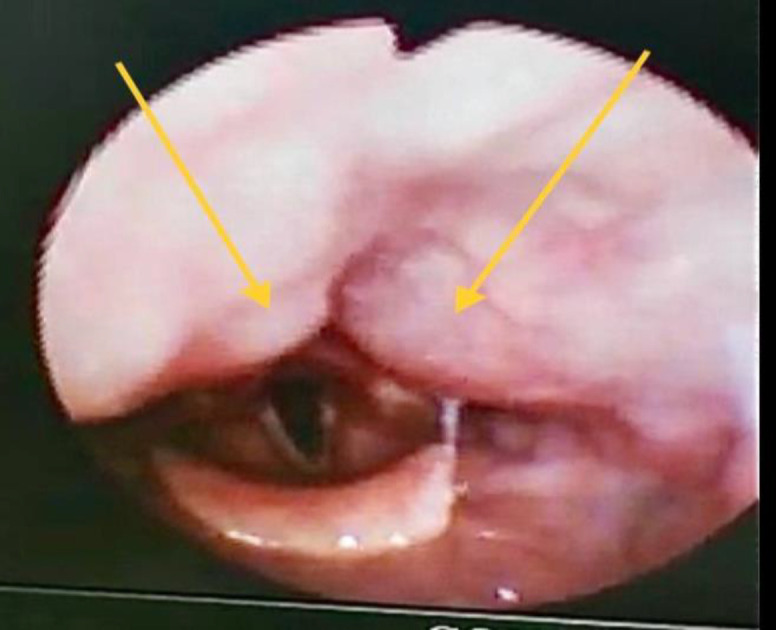
Flexible scope finding of bilateral paramedian retropharyngeal pulsating mass, with normal overlying mucosa (yellow arrow).

On examination, the patient was alert, not septic looking and not kyphotic. The neck and oral cavity examinations were normal. There was no carotid bruit on auscultation. Flexible nasopharyngolaryngoscopy (FNPLS) revealed bilateral paramedian pulsatile mass over the posterior pharyngeal wall, extending from the level of the base of the tongue until the tip of arytenoids. The airway was patent, and the pulsations were synchronous with the radial pulse. Otherwise, the overlying mucosa was normal, while the rest of the laryngeal examination was unremarkable (Figure 1).

**Fig 2:**
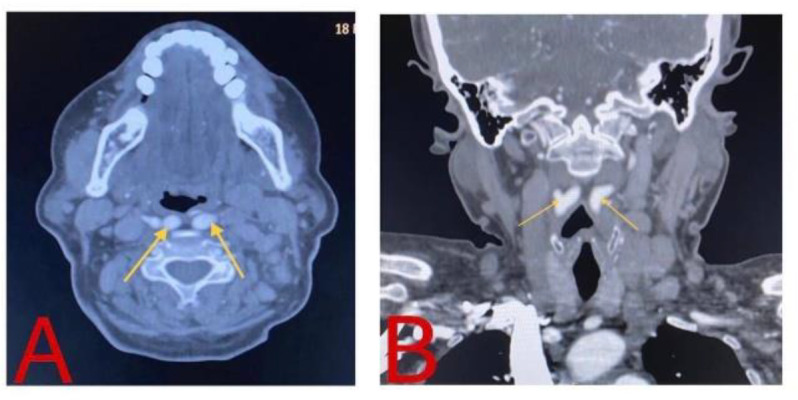
CT angiogram in axial (A) and coronal view (B), showing the medial course of the carotid artery giving the appearance of ‘kissing’ (yellow arrow).

The lateral neck radiograph demonstrated a normal prevertebral space. However, the neck CT and angiogram affirmed an aberrant course of the bilateral carotid artery. Proximally, both common carotid arteries ran within the carotid space up to C5 vertebral level. At the level of C3/C4, the common carotid arteries course in the retropharyngeal space and abut the oropharyngeal wall. Hence, a marked medialization of the left posterolateral pharyngeal wall was demonstrated. The distance between both arteries was only 0.4 cm, causing the carotid arteries are ‘kissing’. Superiorly, both left internal carotid arteries (ICA) and external carotid artery (ECA) ran along the normal course of vascular anatomy. Meanwhile, the proximal segment of the right ICA was coursed along the midline of the C2/C3 vertebra at a distance of 2 cm. Superiorly, both right ICA and ECA were coursed along the normal pathway of the vascular anatomy. There was no aneurysm, intimal flap or arteriovenous malformation of this vessel (Figure 2). The CT findings were explained to the patient. She has been reassured of her condition and subjected to a regular annual follow up.

## Discussion

Anatomical variations of the extracranial internal carotid arteries (ICAs) have a global prevalence of approximately 5%, with a higher incidence among the elderly (2). In many anatomy textbooks, the carotid arteries are described to have a straight course from carotid bifurcation to the base of the skull with branching of ICA and external carotid artery (ECA) at the level of the upper thyroid cartilage in the neck. However, as early as 1868, Henle et al. reported a difference in the course of the carotid artery. Since then, many studies have been done via either post-mortem statistics or advanced angiographic investigation to identify such data. Moreover, Federich et al. recently discovered that among 164 post-mortem subjects, the occurrence of aberrancy was seen slightly more in men (69%) and occurred either unilateral or bilaterally (3). They have categorized the variation into 4 categories using Paulsen et al.’s criteria, i.e.: (i) a straight course if the vertical deviation is less than 15°; 70%, (ii) a tortuosity or S or C-shaped elongation if it deviates more than 15°; 30%, (iii) a kinking of one or more segments if the deviation is between 90° and 145°, and (iv) coiling of the artery for 360° which may cause the appearance of a double loop; with the latter two being less common (less than 7%) and related to older age group (above 50 years old). They also revealed that the kinking and coiling types are frequently associated with the calcification of the vessel (4). Another study by Jens et al. observed that the vessel’s minimum distance to the pharyngeal wall was narrow, ranging from 0.8 to 17.9 mm (mean, 7.0 mm) (2). It is believed that the formation of anatomical variations is due to congenital failure of the dorsal aortic root descends from the third branchial arch during the 5^th^ – 6^th^ embryonic weeks, causing incomplete development, accelerated linear growth and abnormality in the rudimentary stage (5). Several congenital syndromes were associated with these variations, including velocardiofacial syndrome, spondyloepi- metaphyseal dysplasia, and cleft palate (6). These anomalies tend to become more obvious with physiological changes of aging, i.e. loss of vessel wall elasticity (7). Together with atherosclerotic changes or fibromuscular dysplasia, it leads to medial displacement and tortuous calibre of the vessels. Therefore, patient with underlying hypertension or other traditional risks of atherosclerosis has a higher risk of developing this abnormality and its complication (8).

This theory was supported by Zuhal et al., who analyzed the course of the carotid artery in 50 adult cadavers (5). Circumferential sections were obtained, and histological examination of all kinking types of carotid artery specimens showed depleted muscle tissue in tunica media, increased vasa vasorum in the tunica adventitia and loss of integrity in tunica media and tunica adventitia. In addition, the changes in vessel wall structure decrease the elasticity and integrity of the vessel wall, making them more vulnerable to tear or damage (5). Patients with these types of variation are commonly asymptomatic, and finding is often incidental. However, a small percentage of them may present difficulty in swallowing, speech problems (especially in children), foreign body sensation felt in the throat or symptoms of obstructive sleep apnea (3,9). 

They may also present with symptoms of cerebrovascular insufficiency such as neck discomfort, local bruit, hemiparesis, loss of vision, aphasia and seizures (10). 

An anomalous ICA associated with symptomatic cerebrovascular insufficiency has an incidence of 4–16% (11). There should be a high suspicion of an aberrant carotid artery among elderly patients with a kyphotic alignment as the acute cervical fracture may mimic the sign of prevertebral soft tissue swelling, history of trauma or fall should be excluded (1).

Understanding these variations in the neck is essential, as its presence is in close relation to the posterior wall of the pharynx, catastrophic perioperative and postoperative complications can be averted. Otorhinolaryngologists, oral and maxillofacial surgeons must take extra caution, especially during surgery involving or in close proximity to the pharynx, such as tonsillectomy, adenoidectomy, direct laryngoscopy, esophagoscopy or pharyngeal biopsy, transoral tumor resection, peritonsillar abscess drainage and other related oral surgeries. The possible causes of injury can be from suture ligatures, which may pierce and obstruct the blood vessels. This also includes scalpels or biting instruments that may tear vessels and electrical cauterization that might accidentally burn the tissues, as well as the risk of arterial puncture or injection of local anesthetics while conducting the procedures (9, 12). Additionally, a hemorrhage may occur during tracheal intubation. In the hands of an inexperienced surgeon, an asymptomatic patient may be complicated with massive and life-threatening bleeding. This may result in vessel ligation and cerebral infarction, causing neurological deficit (13). In addition, knowledge of its possible variants is also important for head and neck radiologists, taking into consideration that they are responsible for reporting and alerting the surgical team to the relevant findings. Therefore, this aberrancy should be excluded prior to any oro-pharyngeal procedure either via detailed clinical examination or appropriate imaging (14–16).

A suspected carotid artery anatomical aberration can be evaluated with a contrast-enhanced computed tomography (CECT) or CT angiography, which are readily accessible in many health centre (16). A three-dimensional time-of-flight magnetic resonance angiogram with Doppler ultrasonography is also valuable for precise evaluation (4). Once the diagnosis is established, it should be recorded in the patient’s health documentation for future reference and precaution (17). At present, very little data have been reported on the standard gold treatment of such abnormality. Microvascular reconstruction surgery is only recommended by a recent study in cases of oropharyngeal neoplasms and radiologically confirmed retropharyngeal carotid arteries (18). It has also been reported that in symptomatic cases, a surgical correction of elongated carotid arteries is superior to the best medical therapy in preventing stroke (19). However, before any surgical intervention, the risk and benefit must be carefully weighed and tailored accordingly.

In the present case, the patient presented with an acute onset of foreign body sensation felt in the throat. Besides being elderly, the patient neither has other risk factors nor symptoms of complications of such aberrancy. Detailed physical examination and imaging study confirmed the diagnosis and categorized it as a tortuous type with a very narrow distance between the arteries, consistent with the previous prevalence figure. The patient was planning for a watchful follow up with advice. Besides, Lukin et al. reported a gradual migration of the carotid arteries to and from a retropharyngeal position seen in 6.3% of 63 patients (20). Thus, the rationale for a long term follow up (20).

## Conclusion

In conclusion, experiences in the anatomical variants of the carotid arteries are essential before conducting any procedure or surgery to avoid any disastrous complications. Thorough perioperative assessment with accurate imaging techniques and studies are required to evaluate these anomalies in suspicious cases. Given the possible life-threatening complications, surgeons and other medical practitioners should consider the possibilities of these anomalies among their patients. 

Acknowledgement: We thank the Director-General of Health Malaysia for the permission to publish this report on (NIH.800-4/4/1 Jld. 98(49). The author is grateful and acknowledges the valuable contributions of all the helpful co-authors in completing this case report. Verbal informed consent was obtained from the patient. There was no financial funding or conflict of interest to publish the article.
